# Authors' reply

**Published:** 2009

**Authors:** Singh Harsimran, Tewari Anurag, Kaur Balvinder, Garg Suchita

**Affiliations:** 1 Department of Anaesthesiology, Dayanand Medical College and Hospital, Ludhiana, Punjab, India; 2 Department of Anaesthesiology, Dayanand Medical College and Hospital, Ludhiana, Punjab, India; 3 3Department of Paediatrics, Ludhiana Medicity Hospital, Ludhiana, Punjab, India; 4 Department of Anaesthesiology, Dayanand Medical College and Hospital, Ludhiana, Punjab, India

Dear Editor,

I thank the reader[1] for appreciating our indigenous novel method of giving a “U” turn to infusion tubing to increase the negative pressure that would be required to aspirate air.[2] The basic principle of physics behind this technique is that more the length of the “U” more will be the pressure required and hence more the safety. But the length of the infusion tubing is fi xed so we are giving a “U” turn of 15 cm of each side. This length adds safety without need of extension tubing. I agree with the reader that the understanding of basic principles is of foremost importance in practice of intensive care and thank for his interest and appreciation.
